# Intercondylar Notch Impingement of the Anterior Cruciate Ligament: A Cadaveric In Vitro Study Using Robots

**DOI:** 10.1155/2018/8698167

**Published:** 2018-12-10

**Authors:** Ross Wilson, Alan A. Barhorst

**Affiliations:** ^1^Texas Tech University, Mechanical Engineering, Lubbock, Texas 79409-1021, USA; ^2^Alumni Association/LEQSF Professor and Department Head, University of Louisiana at Lafayette, Department of Mechanical Engineering, Lafayette, LA 70503, USA

## Abstract

**Background:**

Research has indicated that a smaller intercondylar notch could cause contact between the anterior cruciate ligament and the femoral notch, which may predispose individuals to an increased rate of anterior cruciate ligament injury.

**Hypothesis:**

Contact between the lateral notch wall and the anterior cruciate ligament does increase the strain past the structural integrity of the ligament.

**Study Design:**

A descriptive laboratory study.

**Methods:**

A biomechanical study using robotic manipulators was conducted to investigate the occurrence of impingement in human cadaver specimens. Six cadaveric knees from six donors (three male and three female) were instrumented with a thin force sensor, placed on the lateral wall of the femoral condyle, and a differential variable reluctance transducer (DVRT) was attached to the middle section of the anterior medial bundle of the ACL. The knees were then moved through a series of flexion (5° to 90°), valgus (0 to 7.5°), and external rotation (0 to 7.5°) movements using two interacting robots.

**Results:**

The results revealed that impingement occurred in both male and female specimens with a maximum impingement force of 28 N. Impingement occurred more prominently in female knees and in the combination loading of valgus and external rotation for both genders. The corresponding strain due to impingement was small or compressive, with the male knee maximum strain less than 1.28% and the female knee strain less than 7.1% in the worse case conditions.

**Conclusion:**

The lack of increased force or strain when impingement occurred indicates that impingement may not affect the healthy function of the knee with a nonstenotic notch. Additionally, the analysis shows that impingement may not be a major contributing factor to anterior cruciate ligament injury, but rather a common occurrence in healthy knees.

**Clinical Relevance:**

Impingement within the femoral notch does not appear to be a major contributory factor to ACL injury. Other more severe injuries to the knee would occur before ACL impingement with the femoral notch becoming a contributing factor to ACL injury. The small sample size limits the conclusivity of the results presented in this research; thus, additional large sample size studies are warranted.

## 1. What Is Known about the Subject

All of ligaments of the knee are vital to healthy knee function; injury to the ACL frequently requires surgery, especially when the ligament experiences complete rupture. There is much debate about the treatment of ligament injuries in the knee, but surgery is not usually required for treatment to repair damage to the PCL, LCL, and MCL. The ACL is injured when the force in the ligament exceeds its mechanical threshold. Patients often complain of hearing a “pop” or “snap” upon injury to the joint followed by pain and swelling shortly afterwards. It is possible to injure the ligament through contact with an outside source, although these types of injury are not as common, with noncontact injuries making up about 70% of injuries to the ACL. Most ACL ruptures occur due to excessive anterior translation or internal rotation of the femur relative to the tibia. Anterior cruciate ligament reconstruction has been identified as a common surgical procedure. In 2004, the American Board of Orthopaedic Surgeons reported that ACL reconstruction ranked sixth among the most common surgical procedures performed by all sports medicine fellows and third among surgeons identified as a generalist [1].

There are a number of postulated mechanisms causing noncontact ACL injury. They are classified relative to the movements in various biomechanical planes of the body. Sagittal plane mechanisms, for instance, anterior shear, are related to forces and moments that are caused by sagittal movements such as flexion of the knee. Coronal plane mechanisms are related to forces and moments that cause coronal movement, and this is valgus movement. Transverse plane mechanisms are related to forces and moments causing traverse plane movements, and these are internal and external rotations. The effect of combined valgus and external rotations may cause increased impingement forces.

In recent years, a few studies have attempted to correlate increased risk for anterior cruciate ligament injury to the geometry of the intercondylar notch. It has been proposed that the ACL could come into contact with the intercondylar notch, thus increasing stress concentrations in the ACL and increasing the risk for injury to the ligament [2, 3]. Studies investigating this hypothesis use MRI scans for visual inspection [4, 5], numerical models to estimate loading [6], and experimental studies measuring loading [7]. Since our experimental study [8], that we have reported herein, this same impingement hypothesis has been investigated relative to gender differences [9] and joint geometry [10]. More numerical studies have also been completed [11]. A comprehensive review of the literature relative to noncontact ACL injuries from 1950–2007 was reported in 2008 [12]. Studies are ongoing relative to impingement after ACL surgery [13–19].

## 2. What This Study Adds to Existing Knowledge

The study conducted provides base-level quantitative data pertaining to the forces induced by the action of impingement of ACL against the intercondylar notch during knee movement. The study was conducted with the aid of two interacting robots in order to readily reproduce various movements causal to ACL injuries. It was found that, as expected, impingement does increase the strain and subsequently the stress within the ACL. However, findings indicate that impingement does not provide enough additional stress in the ACL to be a major factor in ACL injuries.

## 3. Introduction and Background

Most ACL ruptures occur due to excessive anterior translation or internal rotation of the femur relative to the tibia. Anterior cruciate ligament reconstruction has been identified as a common surgical procedure. In 2004, the American Board of Orthopaedic Surgeons reported that ACL reconstruction ranked sixth among the most common surgical procedures performed by all sports medicine fellows and third among surgeons identified as a generalist [1].

Research has indicated that a smaller intercondylar notch could lead to contact between the ACL and the femoral notch which may predispose individuals to an increased rate of ACL injury [3, 20]. It is speculated that contact between the lateral notch wall and the anterior cruciate ligament could increase the strain greater than the structural integrity of the ligament [2, 3, 21]. In recent years, attempts to develop correlations between increased risk for anterior cruciate ligament injury and the geometry of the intercondylar notch have been conducted. Studies involving the ACL and the intercondylar notch have been comprehensive but have failed to address the impingement force and the corresponding change in strain when impingement occurs [2, 3, 18, 22–39].

The ultimate effect ACL impingement has on injury is a subject to debate. Contact between the ACL and the intercondylar shelf as the knee approaches extension and during hyperextension in the joint has been confirmed [40]. In the early twentieth century, impingement on the lateral notch wall using cadaver knees was observed, and it was proposed at the time that the ACL could be subjected to increased load due to this phenomenon [21]. Later, it was proposed that the ACL could come into contact with the intercondylar notch, hence, increasing stress concentrations in the ACL thus increasing the risk for injury to the ligament [2, 3]. Furthermore, it has been theorized that a stenotic intercondylar notch impinges with more force on an ACL than a normal intercondylar notch, thus increasing the risk for injury during cutting and pivoting maneuvers [3]. One study found when cadaver knees were subjected to 30° to 40° flexion and externally rotated past 15°, impingement occurred at the midpoint of the ACL [2].

Sudden tension in the ACL and tibial external rotation combined with a smaller diameter ACL has been proposed to cause increased impingement and stress concentrations in the ACL [20]. It has been proposed that a smaller ACL would have a lower mechanical threshold; studies have shown that the volume of the ACL in the femoral notch is less in women than men relative to height and weight [41, 42]. Others have stated that it has not been determined if notch stenosis is associated with the ACL stretching over the intercondylar notch creating impingement or if a smaller intercondylar notch reflects a smaller ACL [43, 44]. A normal-sized ACL mismatched with a stenotic notch was responsible for injury in patients with narrow notches, but no statistical difference in the notch width index between the genders has been found by at least one study [43].

As evident by the result of these previous studies, there exists a need to better understand the mechanisms causing ACL injuries. The study reported herein provides more quantitative data of impingement forces and strains for consideration by the pertinent medical community. In this work, robots are used to move cadaveric knees in a realistic motion regimes and ACL impingement against the intercondylar notch, and associated forces and strains are measured. The results provided below address the issues raised by previous researchers in this area.

## 4. Methodology

The analysis of the impingement force and the corresponding strain in the anteromedial portion (AM) of the ACL would allow for the comparison of changes in strain during impingement. Therefore, a biomechanical study using robotic manipulators was conducted to investigate the occurrence of impingement in human cadaver specimens and verify if such impingement causes excessive strain. The use of robotic manipulators provides an accurate method to impose and control complex biomechanical movements of the knee joint. Knees were instrumented with contact force sensors and displacement sensors to gather valuable information for analysis relative to ACL injury.

Six fresh frozen cadaver knees, 3 male and 3 female, were procured meeting several criterion. All knees used were from individuals under age of seventy without knee damage or surgery. The donors were also skeletally mature with no history of osteoarthritis. The anthropometric data are summarized in Table 1.

Prior to dissection, the knees were thawed at room temperature overnight. The soft tissue around the knee capsule was removed, but the major ligaments of the knee (ACL, PCL, MCL, and LCL) were left intact for this experiment, except for the outer parts of the LCL, which was removed along with the fibula. The knee was flexed by the hand for a minimum of 20 times through its full range of movements to eliminate the possibility of crimping of ligaments and tendons. Flexing the knee prior to insertion into the robots also conditioned the tissue and removed stiffness from the joint.

**Figure 1:**
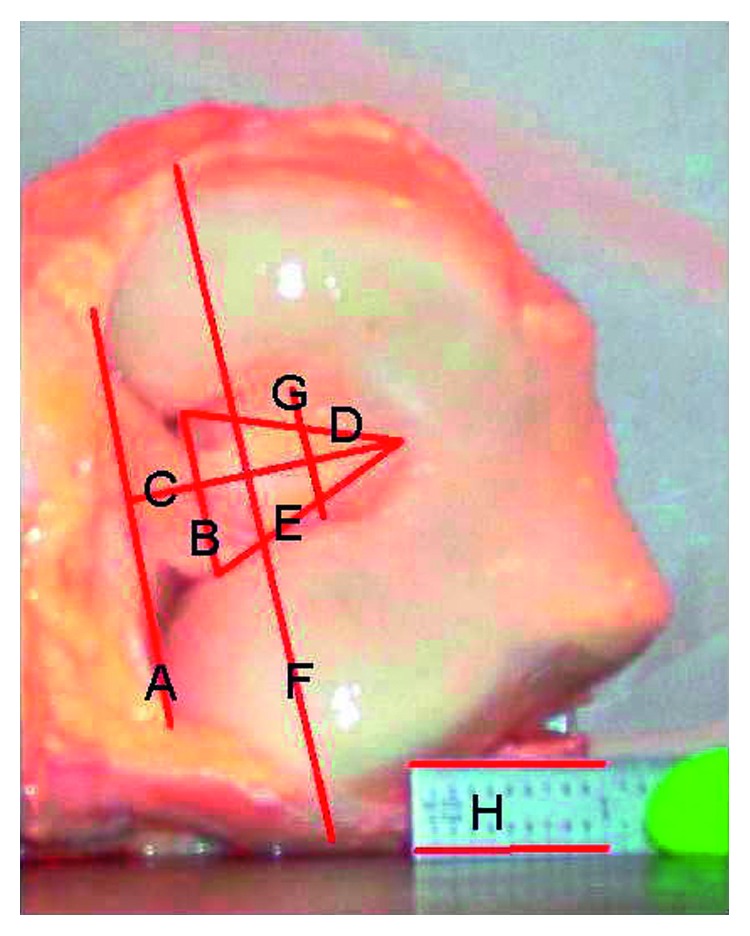
Intercondylar notch measurements: line “C” is the notch height, line “B” is the notch width at the exit, line “F” is the bicondylar width, line “G” is the notch width at the 2/3^rd^ height, and line “H” is the scale.

To relate intercondylar geometry to increased probability of impingement, the geometry of the intercondylar notch was determined with the method prescribed by Anderson et al. [43]. A digital photograph was taken of the knee using an hp 435 photosmart digital camera (3.1 megapixels) (Hewlett Packard, Palo Alto, CA, USA), while the knee was in hyperflexion, along with a reference scale so that the geometry of the notch could be determined. Using Kodak imaging for Windows (Eastman Software Inc., Rochester, New York, USA), line segments were drawn to determine the dimensions of the intercondylar notch and compared with the scale by counting the number of pixels per inch and then comparing that to the number of pixels in each line segment. The measurement of each line segment was made easier by using Screen Calipers 4.0 (Iconico Inc., New York, New York, USA), which allow the user to count the number of pixels in a line segment quickly. The notch width at the exit (NW-E) and at 2/3^rd^ notch height (NW-2/3) were measured by measuring the lengths of line segments “B” and “G” (Figure 1). The bicondylar width (CW) and notch height (NH) were represented by lines “F” and “C.” The notch width indices (NWI-E and NWI-2/3) were defined as the ratio of NW to CW and calculated based on NW-2/3 and NW-E. The anterior cruciate ligament width was also measured using the same photograph, and the minimum width of the ACL was recorded for analysis. Determination of the size of the ACL is important to relate impingement force to the size of the ACL and thus helps to determine what factors are involved in ACL impingement. Tables 2 and 3 summarize this geometry of the specimens as recorded.

### 4.1. Robot and Load Sensor Information

**Figure 2:**
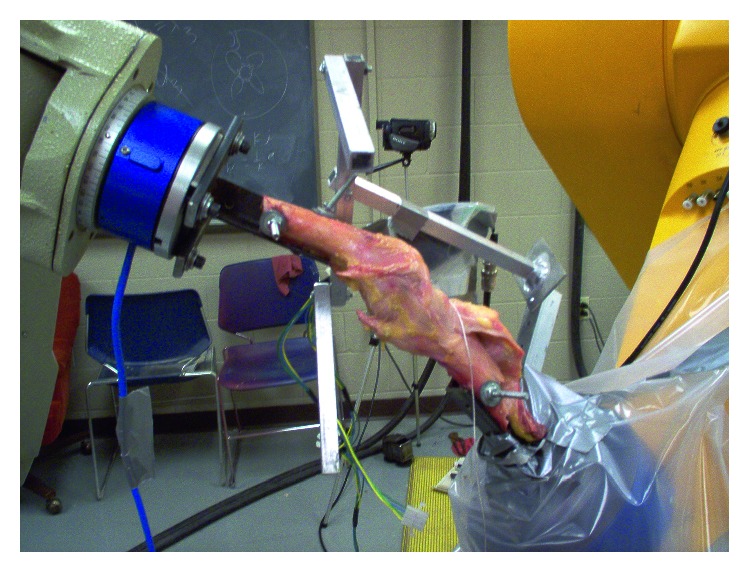
Cadaver knee in custom jigging actuated by two 6-DOF robots; the femur is on the left, and the tibia is on the right.

Two 6-DOF (degree of freedom) robotic manipulators, a Puma model 762 (Unimate, Danbury, CT, USA) and a Staubli model RX-170 (Staubli-Unimation, Duncan, SC, USA), were used in this research (Figure 2). Used in combination, the system allows for movements of both the femur and the tibia. To measure the force each robot exerts on the knee during testing, each robot was equipped with a universal force sensor (UFS), both made by JR3, blue sensors in Figure 2. The Puma robot is equipped with a JR3 UFS (Model 100M40A-U760 100L400, JR3, Woodland, CA, USA), which is accurate to 2% over its full scale. Software developed by Pires [45] is used to collect the data from this UFS. The source code for the software developed by Pires was provided and modified to fit this experiment in order to record data for the specific intervals of time when the Puma robot moves. A custom-made tool, shown in Figure 2, to which the femur is bolted using #10–32 machine screws was used to secure attachment of the bone to the robot. This assures minimal translation of the bone at the attached end. The Puma robot was operated in the position control mode with a repeatability of 0.2 mm. The Staubli robot is equipped with a Model 160M50A-150L950 JR3 UFS and a custom-made tool to which the tibia was bolted also using #10–32 machine screws. The model 160M50A-150L950 JR3 UFS connected to the Staubli robot utilizes Adept's (Adept, Livermore, CA, USA) V+ programming language along with Adept's controller to store and output the load sensor data. The Staubli robot uses built-in UFS software, to accommodate force control movements.

Force control mode works by measuring the force exerted on the tool of the robot, setting limits of force or moments in three coordinate directions, and stopping the robot when the force exceeds the limits. Force control tests on the knee can be used to measure the displacement that the knee undergoes. The Staubli robot can also be operated in the position control mode with an accuracy of 0.04 mm or in the force control mode to an accuracy of 2% at full scale. The UFS attached to each robot measures three forces (Fx, Fy, and Fz) and three moments (Mx, My, and Mz) along an orthogonal axis system. The tool frame of each robot has been orientated such that the translational and rotational movements of the knee correspond to the translational and rotational movements of each robot. The Puma robot can complete flexion-extension, internal-external rotation, and varus-valgus rotation along with translational movements. The Staubli robot has the ability to complete internal-external rotation, varus-valgus rotation, and the translational movements when required, such as performing a simulated Lachman test or simulated joint laxity test. To ensure the movements are accurate, the tool frame of each robot is used along with the controller software to measure changes in rotation and translation. The tool frame allows the robot to rotate and translate about a point at the center of the knee as opposed to the distal end of the tibia or femur, creating a more realistic “roll-glide” movement of the knee. A video of this motion is included in the Supplemental Materials (available here) for this paper.

### 4.2. Descriptions of Robotic Movements

The movements tested attempt to include all normal orientations of the knee, but not extreme enough to cause hyperextension or to simulate injuries through direct loading. During the test, one robot is held static until the other completes movements, but each test takes advantage of utilizing a two-robot system to achieve varus and valgus torque along with internal and external rotations created by both the femur and the tibia. The use of a two-robot system allows for comparisons of orientations created by the movement of the femur and the tibia. An example of this is loading the knee to 5° valgus by rotating the femur 5° in the valgus direction without moving the tibia and then comparing this to rotating the tibia 5° valgus while holding the femur static. This system attempts to more accurately predict injuries to the knee by moving both the femur and the tibia, instead of having a single robot and keeping either the femur or the tibia fixed to a base. The movements are shown in Table 4: knee movements, including angles of internal and external rotation, angles of varus and valgus rotation, and flexion angle.

The Staubli robot also performs a simulated Lachman test and simulated joint laxity test by operating in the force control mode. The Staubli robot records the initial location of the tibia and moves perpendicular to an axis parallel to the surface of the tibia, in the anterior direction, until it records 100 N force for a Lachman test. For a joint laxity test, the Staubli robot translates along the same axis as before, but with a posterior translation. Once 100 N of force for a Lachman test or 120 N of force for a joint laxity test has been applied to the knee, the Staubli robot arm stops and records the location, so the displacement can be measured between the start position and end position of the test, and this information is recorded in a file for analysis. All of this is completed, while the Puma robot holds the femur fixed at 20° flexion. These data could be compared later with magnetic resonance imaging (MRI) of the knees for differences in tibia plateau to determine the consistency of the Lachman and joint laxity tests as an indicator of damage to the ACL.

### 4.3. FlexiForce® Sensor, DVRT Sensor, and LabView Setup

**Figure 3:**
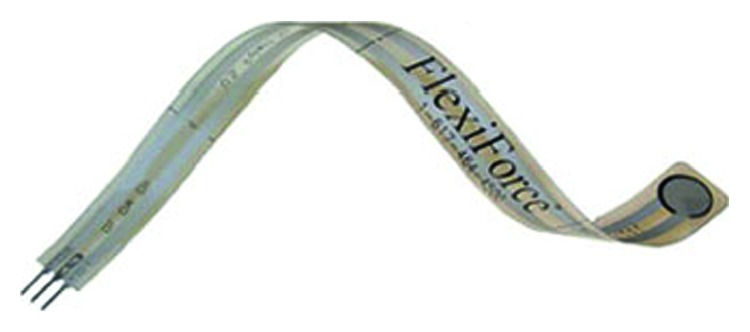
FlexiForce® sensor.

To measure the force between the anterior cruciate ligament and the lateral notch wall, a force-sensing resistor (FlexiForce®, Tekscan, Boston, MA, USA) is used, and the device is shown in Figure 3. The device is inserted between the ACL and attached to the lateral notch wall. The FlexiForce® sensor has the ability to measure force between the two surfaces.

In this application, the model A201-25 sensor was used. It had a length of 8 in. (203 mm), a thickness of 0.008 in. (.208 mm), a sensing area of 0.375 in^2^ (9.53 mm^2^), and a maximum loading capacity of 25 lbs (111 N). The active sensing area is shown in Figure 3 as the silver circle on the end of the sensor. The typical performance of the sensor is presented in Table 5.

**Figure 4:**
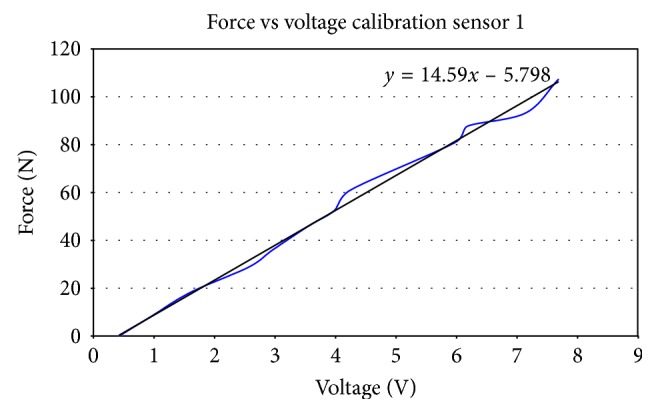
Calibration of the FlexiForce® sensor.

**Figure 5:**
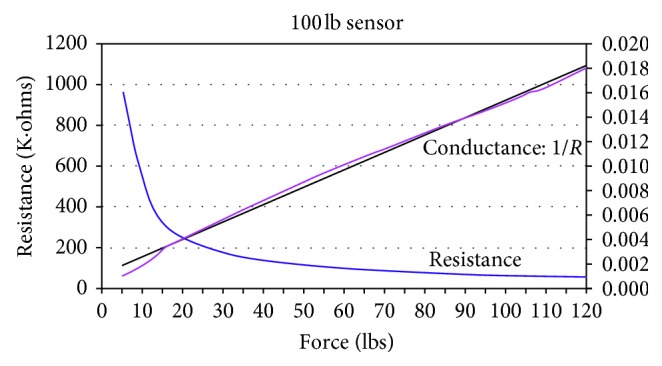
Force, resistance, and conductance relationship for the FlexiForce® sensor (http://www.tekscan.com/pdfs/FlexiForceUserManual.pdf).

A conditioning circuit (appendix A) allows for a linear relationship between force and voltage after the sensor has been conditioned and calibrated.  Figure 4 shows the calibration graph of one of the three sensors used during testing. The measured relationship between force, resistance, and conductance is shown in Figure 5. It is important to note that the use of a conditioning circuit in this study provides more accurate and repeatable measurements.

In order to reduce the noise in the signal, a filter was added to the conditioning circuit of the sensor. The low-pass filter consists of a 10 Ω resistor connected to the voltage output (going to the LabView DAQ board) and a 470 *μ*F capacitor, which was connected to the ground. This filter provides a cutoff frequency of 34 Hz. In addition, the filter required the modification of the recommended conditioning circuit. A smaller resistor of 0.47 Ω was installed in place of the recommended 1 KΩ resistor.

The sensor was attached to the condyle using the thin plastic film on each side of the sensor, which was taped to the sensor using a cellophane tape. Attached to both sides of the sensor was a thin piece of aluminum, commonly called a “puck.” The “puck” is 0.015 in. (0.381 mm) thick. The addition of two “pucks,” the thin plastic film, the sensor, and the tape to the lateral notch wall reduce the width of the notch by approximately 1.5 mm. This piece allows for the load applied by the ACL to be more accurately measured. The “puck” provides an even distribution of the loading on the sensor, by preventing the ACL from coming into contact with the edge of the sensing area and reducing stress concentrations applied to the sensor. In addition, onto the side that contacts the lateral notch wall, the sensing edge will not come into contact with the bone, altering the force measured by the sensor. The sensor and its conditioning circuit are connected to data acquisition equipment. LabView 8.0 (National Instruments, Austin, Texas, USA) was used to collect data from the FlexiForce® sensor and DVRT. The LabView DAQ board model number is NI PCI-6042E. The use of LabView and the digital input/output from the robotic controllers allowed for triggering of the DAQ system to record information from the FlexiForce® sensor and DVRT only when one of the robots moved.

A differential variable reluctance transducer (DVRT) (DVRT, MicroStrain Inc., Burlington, VT, USA) was inserted in the AM bundle of the ACL to measure the strain in the ligament during loading. This sensor was connected to LabView through a conditioning circuit provided by the manufacturer. A low-pass filter in the LabView DAQ software was set with a cutoff frequency of 3 Hz to filter noise. The DVRT was calibrated by determining the reference length of the sensor relative to the voltage output of the sensor. The initial position of the knee was used with the calibration to define the reference length used to calculate strain. The DVRT used was designed for orthopaedic research. Two small barbed prongs are located on the sensor for attachment to the ACL, specifically to ensure that the sensor does not become dislodged during movements. The steps used to collect data via LabView was similar to the FlexiForce® sensor; the collection of data was properly timed with the digital input/output of the robotic controllers to only measure displacement during robotic loading of the knee specimen.

### 4.4. Robot Control Circuit

To properly time the movements, digital input/output (I/O) signals from each robot were required. The digital circuit allows for the robots to be “aware” of each other's movements. Each move was properly timed with the data acquisition system and the movement made by the other robot. Each robot was equipped with two input and two output signals. A schematic and description are provided in the Appendix section.

## 5. Results

The mechanisms of injury to the anterior cruciate ligament are extremely complex and still not fully understood. This work focuses on the possibility of ACL injury due to impingement with the lateral notch wall. The impingement force compared with the force measured by the UFS during impingement will shed light on the significance of impingement as a cause of ACL injury. The measurements of strain in the AM bundle of the ACL will determine if impingement increases the strain in the ligament.

Impingement was observed in five of the six knees tested in this study. Five of the six knees experienced impingement under combined loading of valgus and external rotation. Impingement was measured in two specimens, both female, during pure valgus movement. During pure external rotation, impingement occurred in three specimens, two female and one male.  Table 6 provides a summary of the flexion angles when impingement was measured and the corresponding position of the knee.

### 5.1. Strain and FlexiForce® Sensor Measurements under Pure External Rotation

**Figure 6:**
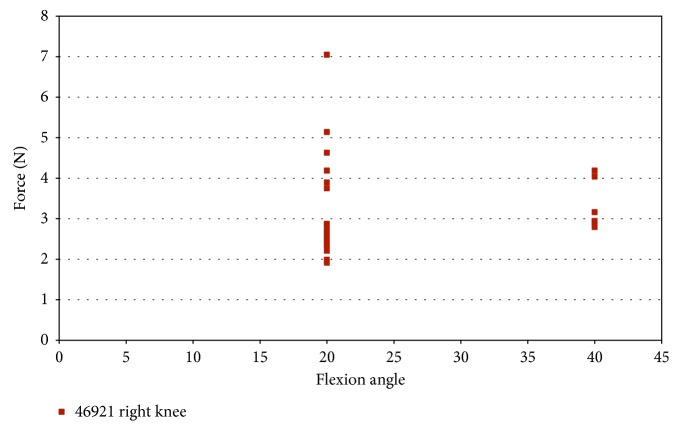
Impingement force measured with the FlexiForce® sensor vs flexion angle; external rotation in the male specimen (46921).

**Figure 7:**
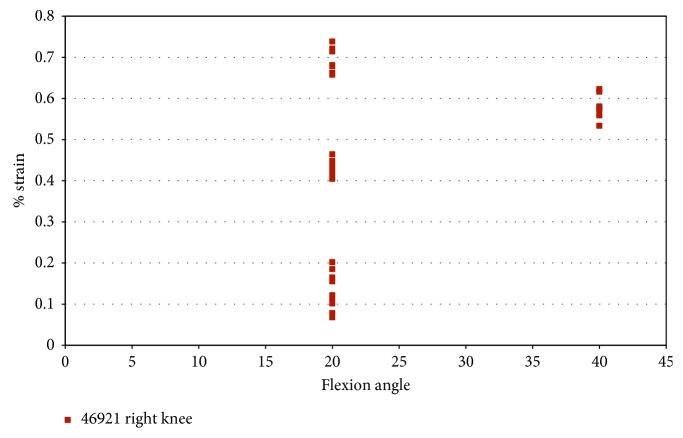
Strain during impingement measured with DVRT vs flexion angle; the external rotation in the male specimen (46921).

In specimen 46921, impingement was measured at 20° and 40° flexion during pure external rotation.  Figure 6 indicates that the maximum impingement force was approximately 7 N at 20° flexion and approximately 4 N at 40° flexion. Impingement at 20° flexion indicates more contact than 40° flexion.  Figure 7 shows the strain measured during impingement versus flexion angle for pure external rotation. The AM portion of the ACL during pure external rotation, in specimen 46921, experiences less than 0.75% strain at 20° flexion and less than 0.65% at 40° flexion.

**Figure 8:**
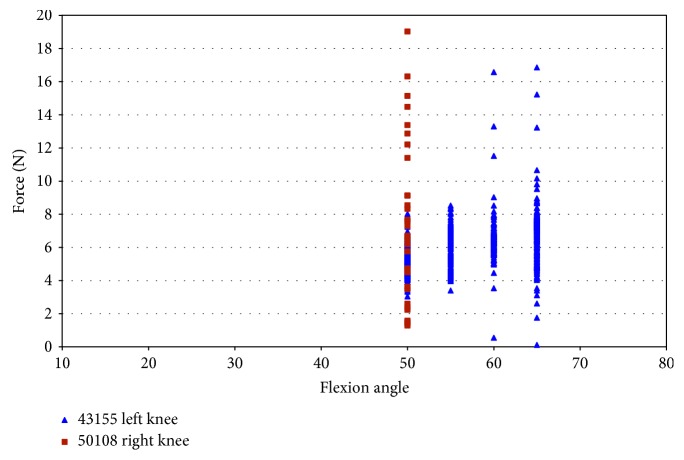
Impingement force measured with the FlexiForce® sensor vs flexion angle; external rotation in female specimens.

Impingement was also recorded in two female specimens during pure external rotation. Impingement force was observed between 50° and 65° of flexion with maximum magnitudes of 19 N for specimens 50108 and 16.86 N and for specimen 43155 (Figure 8). Specimen 50067 did not show any evidence of impingement during pure external rotation. Impingement forces in specimen 43155 occurred from 50° to 65° of flexion (Figure 8), with the largest impingement forces (17 N) occurring at 60° and 65° flexion. Impingement was only measured at 50° flexion during pure external rotation in specimen 50108. The impingement force varied more in specimen 50108 than in specimen 43155, and the magnitude of force was greater (19 N).

**Figure 9:**
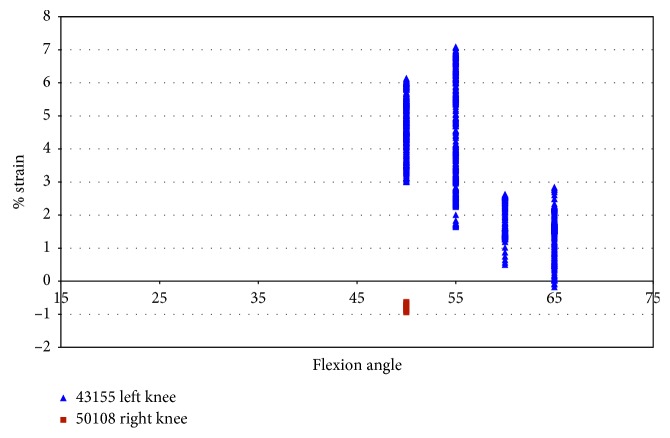
Strain during impingement measured with DVRT vs flexion angle; external rotation in female specimens.

In specimen 43155, during impingement, the strain increased from −0.5% to 6.9% in the AMB of the ACL, under pure external rotation of 2.5° to 7.5°. The largest level of strain was at 50° and 55° flexion, and strain decreased with an additional increase in the flexion angle (Figure 9). The strain in the ACL (43155) decreased after 55° flexion, while the maximum force of impingement increased. In specimen 50108, the strain was compressive with little variation.

**Figure 10:**
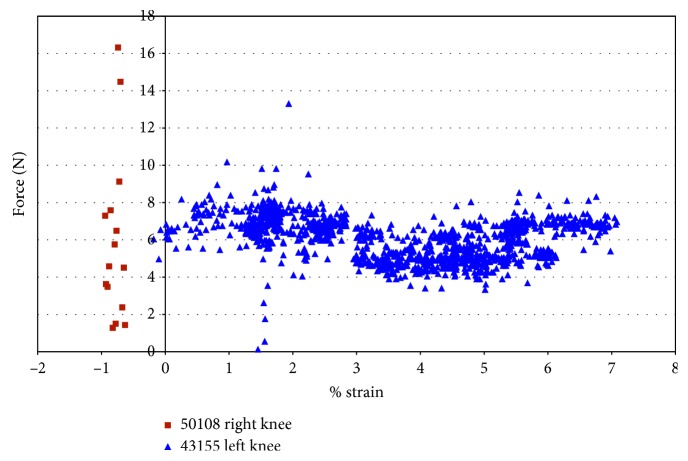
Impingement force measured with the FlexiForce® sensor vs strain during impingement measured with DVRT; external rotation in female specimens.

For knee 50108, despite little variation in strain, the impingement force varied from approximately 1 N to 16 N (Figure 10). The filter in LabView used with the DVRT did not allow the strain to be measured within the first 0.2 seconds at the start of movement. Thus, some of the impingement forces are not shown in the force versus strain plots. Knee 43155 had more points of impingement and a larger range of strain, but lower impingement forces (Figure 10). Figure 10 also shows that the maximum strain, in 43155, occurred during a measured 7 N impingement force, and the maximum impingement force (13 N) occurred when the measured strain in the AMB of the ACL was less than 2.0%. In specimen 43155, the impingement force is consistently between 6 N and 8 N, while the strain ranges from 0.35% to 2.75%; then, the forces decreased to a range of 4 N to 6 N with the strain ranging from 3.0% to 5.0%; finally, the forces were approximately 6.5 N, while the strains ranged from 5.25% to approximately 7%.

### 5.2. Strain and FlexiForce® Sensor Measurements under Pure Valgus Rotation

**Figure 11:**
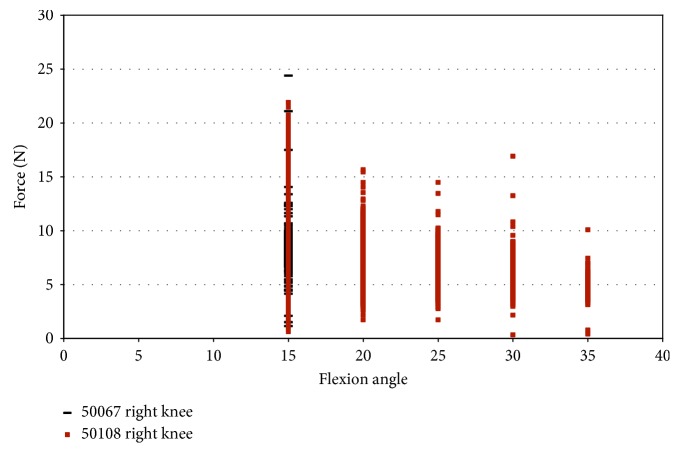
Impingement force measured with the FlexiForce® sensor vs flexion angle; valgus rotation in female specimens.

Loading the knee in pure valgus from 2.5° to 7.5° produces different results regarding impingement forces. The strain measurements were similar to those measured during pure external rotation, but impingement was measured over larger ranges of flexion. Under pure valgus loading, impingement was measured in two female knees (50067 and 50108). Impingement was not measured in the female donor 43155 during pure valgus rotation and was not measured in any male donors. An important note is that, during pure external rotation, in specimen 50108, impingement was measured at 50° flexion, while during pure valgus rotation, impingement was measured over a range of 15° to 35° of flexion (Figure 11).

Specimen 50067 experienced impingement at 15° flexion with a maximum magnitude of 24.39 N under pure valgus movement. Impingement forces in specimen 50108 ranged from 10 N at 35° flexion and a maximum of 21.89 N at 15° flexion. The impingement force decreases with an increase in the flexion angle, and impingement was not experienced after 35° of flexion (Figure 11).

**Figure 12:**
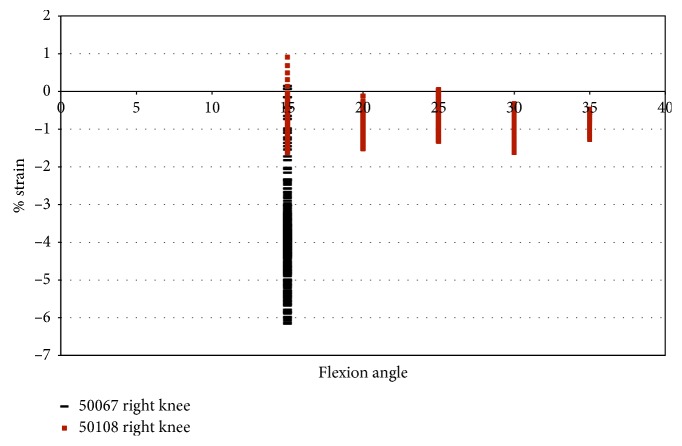
Strain during impingement measured with DVRT vs flexion angle; valgus rotation in female specimens.

Knee 50067 had a maximum strain of less than 0.2%. In donor 50067, almost all of the strains were compressive (Figure 12). It should be noted that specimen 50067 was an abnormally small knee, and during the testing process, the notch roof interfered with the DVRT. Since impingement occurred when the knee was near full extension, some of the strain measurements, particularly those less than −4.0 %, are due to the DVRT contacting the roof of the notch.

Knee 50108 had a maximum strain of 0.91% at 15° flexion. During impingement in 50108, under pure valgus loading, the strain was observed to be mostly compressive despite a decrease in impingement force.

**Figure 13:**
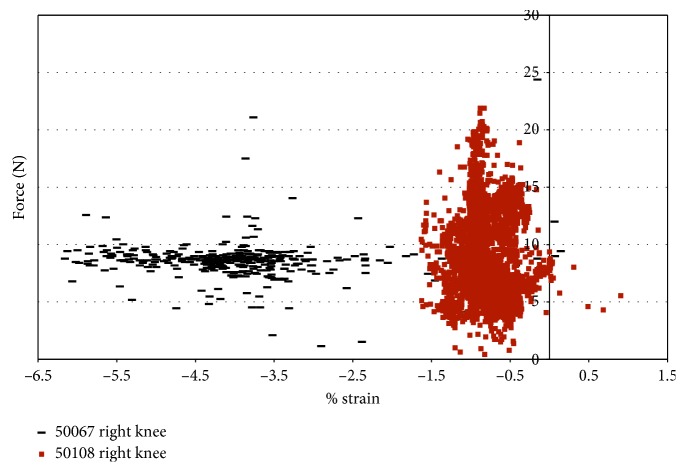
Impingement force measured with the FlexiForce® sensor vs strain during impingement measured with DVRT; valgus rotation in female specimens.

The maximum force of impingement in specimen 50067 occurred when the anteromedial bundle of the ACL was in compression (Figure 13). For knee 50067, the impingement forces were approximately 9 N, with compressive strain (Figure 13). Figure 13 indicates that, in specimen 50108, the two largest impingement forces occurred in compressive strain. Most strains were compressive in specimen 50108. During pure valgus movement, it appears impingement does not affect the strain in the AM portion of the ACL.

### 5.3. Strain and FlexiForce® Sensor Measurements under Valgus and External Rotation

Under combination loading of valgus and external rotation, impingement was measured in five of the six specimens tested. All the female specimens and two male specimens experienced impingement during combined movement. The impingement forces were spread over a wide range of flexion angles in male specimens, while female specimens experienced impingement more at specific flexion angles.

**Figure 14:**
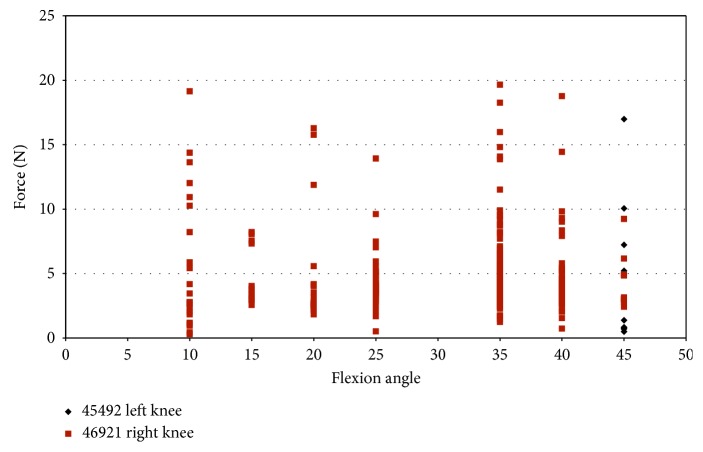
Impingement force measured with the FlexiForce® sensor vs flexion angle; combined loading valgus and external rotation in male specimens.

Impingement in knee 46921 was spread over a larger range of flexion angles (10°–45°) for a combined loading of 2.5°–7.5° valgus and 2.5°–7.5° external rotation (Figure 14). The maximum impingement force was less than 20 N (Figure 14), which occurred at 35° flexion. Specimen 45492 experienced little impingement in combined movement, which occurred at 45° flexion with a maximum magnitude of 16.99 N (Figure 14). In specimen 45492, most of the impingement forces are less than 5 N.

**Figure 15:**
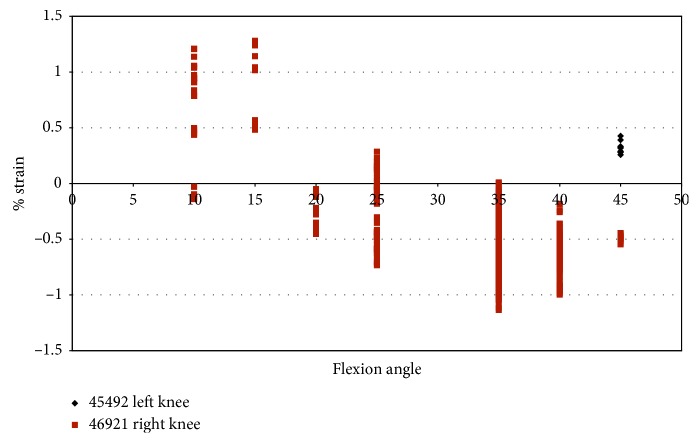
Strain during impingement measured with DVRT vs flexion angle; combined loading valgus and external rotation in male specimens.

Strain measurements for male specimens, shown in Figure 15, indicated that the maximum strain on the anteromedial bundle during impingement was less than 1.3%. The strain during impingement for specimen 46921 is above 1.0% for flexion angles 10° and 15° and then becomes mostly compressive with a continued increase in flexion angle (Figure 15). Specimen 45492, which had a maximum impingement force of 16.99 N, experienced strains less than 0.5%.

**Figure 16:**
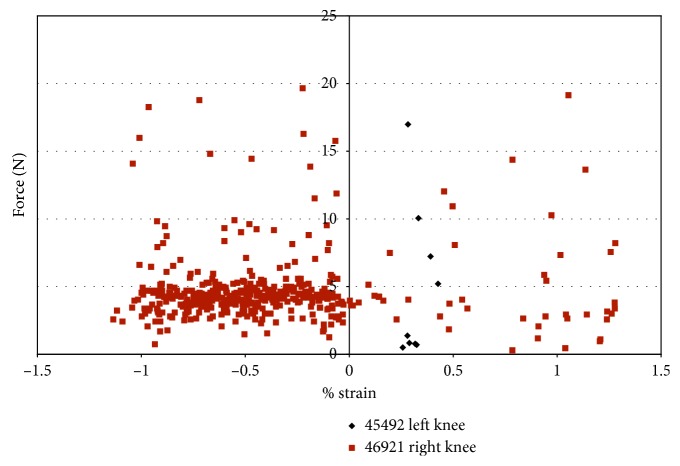
Impingement force measured with the FlexiForce® sensor vs strain during impingement measured with DVRT; combined loading valgus and external rotation in male specimens.

The impingement force was plotted against the strain during impingement (Figure 16). In Figure 16, most of the forces of impingement are approximately 5 N, in specimen 46921, with compressive strain. The largest impingement force occurs when there was compressive strain on the ACL in specimen 46921.

Also when comparing the impingement force against the strain, the largest impingement forces, for specimen 46921, occurred from −1.1% to 1.2% strain. Most strain measurements were when the AM bundle of the ACL was in compression, and the impingement force was less than 5 N. Strain measurements in specimen 45492 were less than 0.5% with little variation, and the impingement force ranged from approximately 0.1 N to 16.99 N.

**Figure 17:**
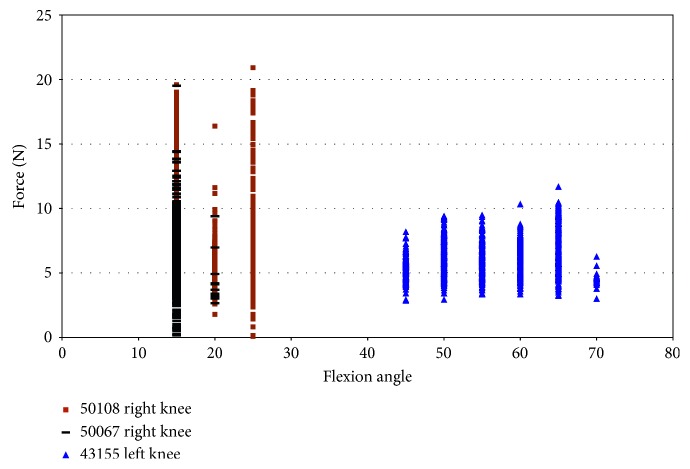
Impingement force measured with the FlexiForce® sensor vs flexion angle; combined loading valgus and external rotation in female specimens.

Under combined valgus and external rotation, impingement was observed in all female specimens. The combination of 2.5° to 7.5° valgus and external rotation of 2.5° to 7.5°, in female specimens, created the largest level of impingement. Specimen 50108 and 50067 experienced impingement at 15° to 25° of flexion, while 43155 experienced impingement between 45° and 70° of flexion. In comparison, during pure external rotation, impingement was measured at 50° flexion in specimen 50108 and from 50° to 65° of flexion in specimen 43155. Also, considering under pure valgus loading in specimen 50067, impingement was measured at 15° flexion and from 15° to 35° of flexion in specimen 50108. Specimen 50108 had a maximum impingement force of 20.91 N at 25° flexion; specimen 50067 had a maximum impingement force of 19.5 N at 15° flexion; and specimen 43155 had a maximum impingement force of 11.67 N at 65° flexion (Figure 17).

For specimen 50108 and 50067, impingement occurred at a lower range of flexion. In 50067, impingement was only measured at 15° flexion. While in 43155, the impingement forces were spread over a larger range of flexion angles, but with lower impingement forces. The flexion angles impingement occurred under pure valgus and pure external rotation were similar for specimen 50067 and 43155, but during external rotation loading in 50108, impingement was measured at 50° flexion. Also during valgus, loading impingement was observed, in specimen 50108, during 30° and 35° of flexion, but not under valgus and external rotation.

**Figure 18:**
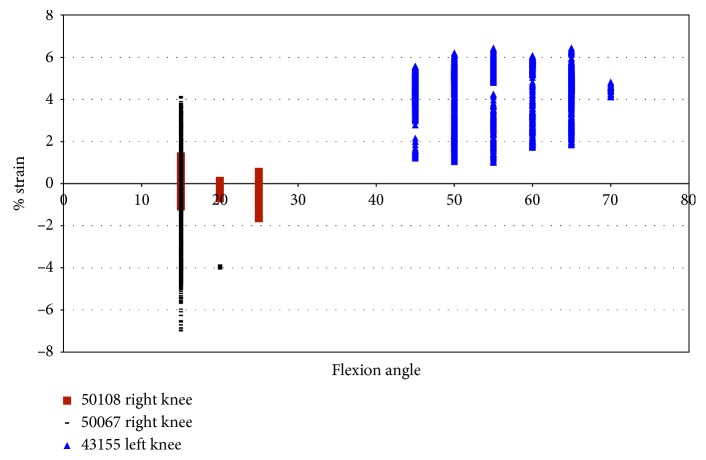
Strain during impingement measured with DVRT vs flexion angle; combined loading valgus and external in female specimens.

The measured impingement forces during combined loading of female knees were similar to that of the male knees. However, Figure 18 indicates that the female specimens experienced an increase in strain. The maximum strain in the AM bundle of the ACL during impingement for specimen 50108 was 1.5% at 15° flexion. For specimen 50067, the maximum strain during impingement was approximately 4% at 15° of flexion. Specimen 43155 experienced 6.3% strain during impingement at 55° and 65° of flexion.

**Figure 19:**
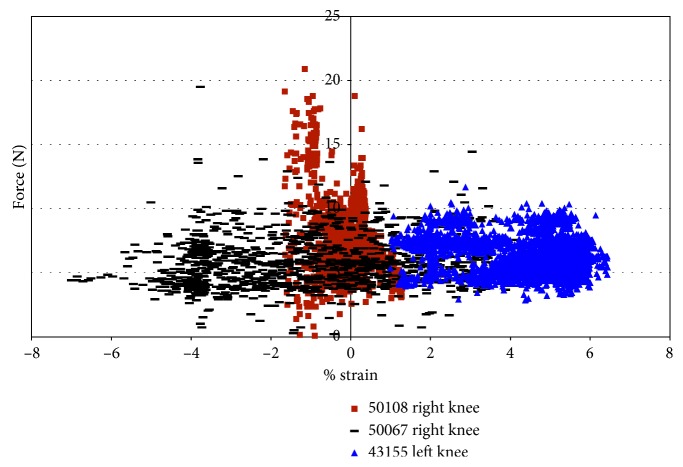
Impingement force measured with the FlexiForce® sensor vs strain during impingement measured with DVRT; combined loading valgus and external rotation in female specimens.

Plotting force of impingement versus strain allows examination of any existing correlations (Figure 19). For specimen 50108, the force of impingement increases at strains of approximately −1% and 1%. A maximum impingement force of 20.91 N occurs with the anteromedial portion of the ACL in compression (Figure 19). The only correlation, for specimen 50108, is that the force of impingement increases when the strains in the AMB of the ACL were at 0.3% and −1.0% (Figure 19). Specimen 50067 experienced impingement forces of approximately 5 N with the corresponding strain ranging from −7.0% to 3.5%. The maximum impingement force, 19.5 N, occurred during compressive strain. This specimen had a large range of strain values with fairly constant impingement forces, although the larger range of strain values may be due to contact between the DVRT and the notch roof. Specimen 43155 experienced more impingement forces with the AMB of the ACL in tension. The strains ranged from approximately 0.75% to approximately 6.5%, and the corresponding impingement forces are approximately 5.8 N. The quantity of points occurs when the impingement forces were approximately 5.3 N, and the strains were greater than 4%. Specimen 43155 shows disparity from the other specimens studied. The disparity was that the impingement forces were lower, but the measured strain was significantly higher than any other specimen tested.

### 5.4. Discussion of Impingement Force and Strain

The male knees experienced fewer points of impingement, meaning impingement occurred in shorter time intervals, but the impingement forces measured are similar in magnitude to female specimens. One male knee (45492, left knee) experienced little impingement, and it occurred in a combined loading of 2.5°–7.5° valgus rotation and 2.5°–7.5° external rotation at 45° flexion with a maximum magnitude of 16.99 N. The strain measurements indicated that the AM portion of the ACL experienced less than 0.5% strain. A second male knee (50137, left knee) experienced no impingement in any of the loadings. Finally, the third male knee (46921, right knee) had a noticeable amount of impingement with a combined loading of 2.5°–7.5° valgus and 2.5°–7.5° external rotation with a maximum magnitude of 19.65 N. In the abovementioned specimen, a small amount of impingement was measured during pure external rotation during 20° and 40° of flexion with a maximum magnitude of 7.05 N. Impingement was not measured in any of the male specimens during 2.5° to 7.5° valgus movement. The female knees experienced more sustained impingement and impingement with pure external and pure valgus rotation. The impingement forces in female donors occurred at lower flexion angles, unlike the male knee (46921) which was spread over a larger flexion range. During pure external rotation from 2.5° to 7.5°, impingement was detected in specimen 50108 (female, right knee) and 43155 (female, left knee). During pure valgus rotation between 2.5° and 7.5°, impingement was measured in specimen 50067 (female, right knee) and 50108. In the combined loading tests, when subjecting the specimen to 2.5° to 7.5° valgus along with 2.5° to 7.5° external rotation, impingement was measured in all female knee specimens.

During pure external rotation, there was not a clear trend of increased strain in the AMB of the ACL with increased impingement force. During pure external rotation, specimen 50108 was not affected by impingement, since all impingement occurred when the AM portion of the ACL was in compression. In donor 50108, the strain did not show any noticeable change while the force of impingement ranged from less than 2 N to 19.3 N. Specimen 43155 had lower measured impingement forces than 50108 but showed an increase in strain. These results could indicate that the ACL of some individuals is affected by impingement. There was no relationship between increased strain and increased force of impingement. It is possible that the elevated strain values were a characteristic of this knee (50108) and not an indicator that the impingement force increased the strain in the ACL.

The female specimens have a greater disparity in strain measured and impingement force compared to the male specimen during pure external rotation. The male specimen (46921) had less than 1% strain in the anteromedial bundle of the ACL when impingement was measured during external rotation. The results of the abovementioned male specimen and female specimen 50108 are similar, but 43155 showed a increase in strain during these movements. The magnitude of force during pure external rotation was much greater in female specimens. The female specimens had maximum impingement forces of 16.3 N (43155) and 19 N (50108). The male specimen, 46921, had a maximum impingement force of 7 N.

In male specimens, impingement does occur and was measurable, but the force of impingement was not significant. Most of the impingement forces are less than 5 N, and during impingement, the strain on the AM bundle of the ACL never exceeds 1.3%. There was not a definitive relationship between an increase in strain and an increase in impingement force for the male specimens tested. The strain measured during combined valgus and external rotation shows an increase in strain, in female specimens, when impingement occurred. Pure valgus rotation resulted in higher impingement forces in female specimens than combined loading, but lower strain.

**Figure 20:**
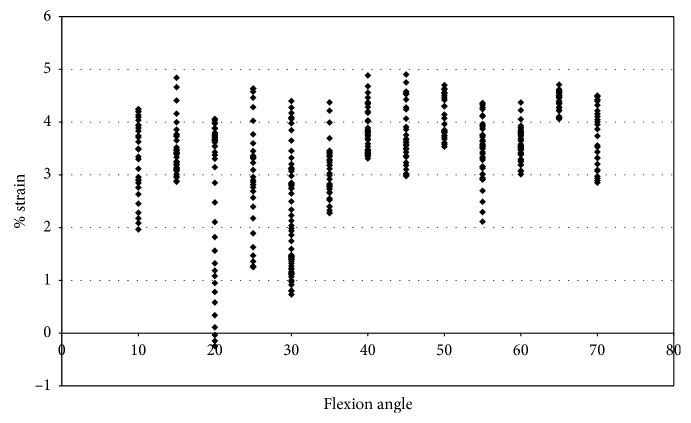
Strain measured with DVRT with no measured impingement vs flexion angle; valgus rotation 43155.

Specimen 43155 had a higher increase in strain than the other specimen tested. In order to determine if impingement was responsible for the increase in strain or if movement was causing the increase in strain, the strain was plotted against the flexion angle under pure valgus rotation (Figure 20). When comparing Figure 20, Figure 18, and Figure 9, it can be determined that impingement could only account for as little as a 1% to 2% increase in the maximum measured strain. It is important to consider that these were different movements. The knee will behave differently during different movements, and thus impingement may not contribute to any increase in strain.

**Figure 21:**
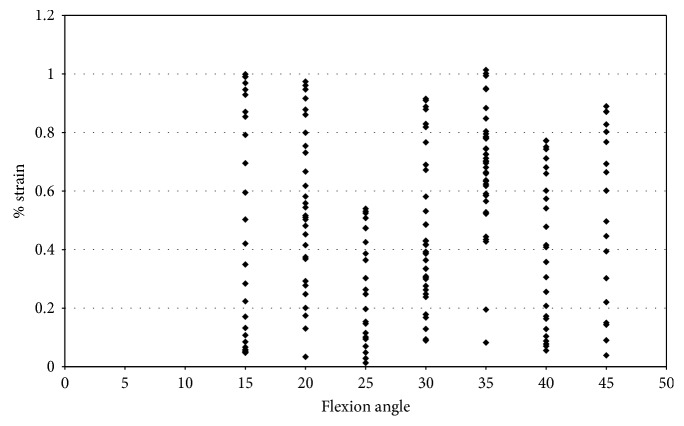
Strain measured with DVRT with no measured impingement vs flexion angle; external rotation 50067.

Specimen 50067 did not have impingement in pure external rotation.  Figure 21 shows the strain in specimen 50067 during pure external rotation. Specimen 50067 has less than 1% increase in the maximum strain measurements taken during pure valgus rotation and pure external rotation. Specimen 50067 had less than a 3.0% increase in the maximum strain measured during combined movement compared to pure external rotation.

During combined loading of valgus and external rotation, female knees experienced impingement forces on the same magnitude as male knees under the same loading. The strain in male knees did not exceed 1.3% during combined movement. In female knees during combined movement, strains exceeded 6% in specimen 43155, and every female knees experienced strains greater than 1.3%. Despite a lack of correlation between an increase in impingement force and an increase in strain in female knees, the amount of strain the AMB of the ACL undergoes provides a significant result. It is possible that impingement does not affect the function of the ACL and is only a normal attribute of valgus and external rotation movements. It is also possible that impingement between the lateral notch wall and the anterior cruciate ligament has a slight affect on the injury of the ACL in female knees.

### 5.5. JR3 Universal Force Sensor Measurements under Pure External Rotation

The JR3 universal force sensor attached to the Staubli robot was used to measure the force exerted by the robots on the given cadaver knees. Recording only one force sensor was required because only one robot moves at a time, and all of the movements in which impingement is measured (valgus and external rotation) are made by the Staubli robot. The magnitude of force on the UFS was plotted against the flexion angle to determine if impingement could affect the force on the knee. It is important to consider the specimens are subjected to rotational movements (valgus and external rotation), so the magnitude of the torque is very important to consider along with the magnitude of force on the knee during the prescribed range of movements. The range of impingement force, range of strain during impingement, and range of net force and torque measurements taken by the JR3 UFS are tabulated. The tabulated data did not show any correlation of increased force on the knee during impingement or insignificant strain during impingement.

Knee 46921 experienced impingement during 20° and 40° of flexion, under pure external rotation.  Table 7 shows the strain during impingement, impingement force, range of JR3 UFS net force, and moment measurements at flexion angles where impingement was measured.  Table 7 indicates that the net force and moment on the knee are significantly larger than the impingement force. The JR3 force and moments data were plotted against the flexion angle. The range of net force and torque measured are given, but it is important to consider that impingement occurs near the extremes of motion, and the direct comparison of impingement force should be made to the maximum net force and torque during the loading.

Specimen 50108 experienced impingement at 50° flexion. The strain in the AM portion of the ACL was compressive. Presented in Table 8 is the range of impingement forces, so they can be compared with the range of forces measured by the UFS during impingement. The AM portion of the ACL was in compression during impingement, and this indicates that the force and torque measured by the JR3 UFS were due to the resistance of the knee during external rotation and not the impingement force measured.

**Figure 22:**
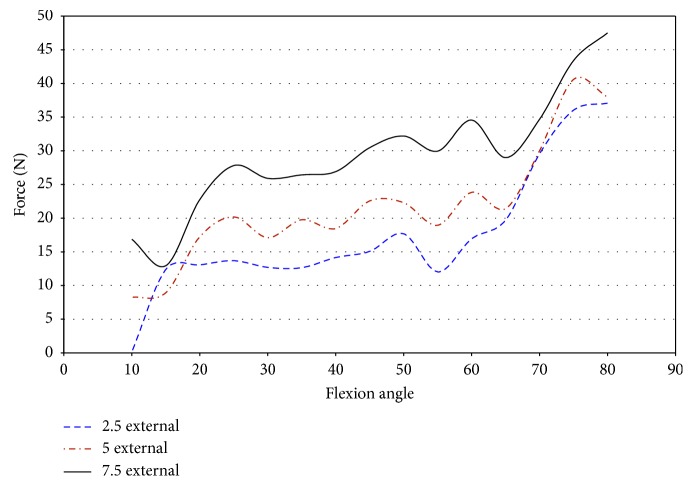
Magnitude of force measured with JR3 UFS vs flexion angle; external rotation 43155.

In specimen 43155, impingement was measured between 50° and 65° of flexion. Since higher strains were found in specimen 43155, the entire range of net force and torque with respect to the flexion angle was plotted to determine if impingement could affect the force of loading.  Figure 22 shows that the force on the knee ranged from 12.0 N to 34.5 N, while impingement was measured. In this knee, the impingement force was significantly lower than the maximum magnitude of force on the knee when subjected to 7.5° external rotation. There exists a significant difference between the forces measured at 7.5°, 2.5°, and 5° external rotations, but this disparity was not isolated to the flexion angles when impingement occurred. The change in force for 7.5° external rotation does not indicate a change in force measured by the UFS relative to ACL impingement.

**Figure 23:**
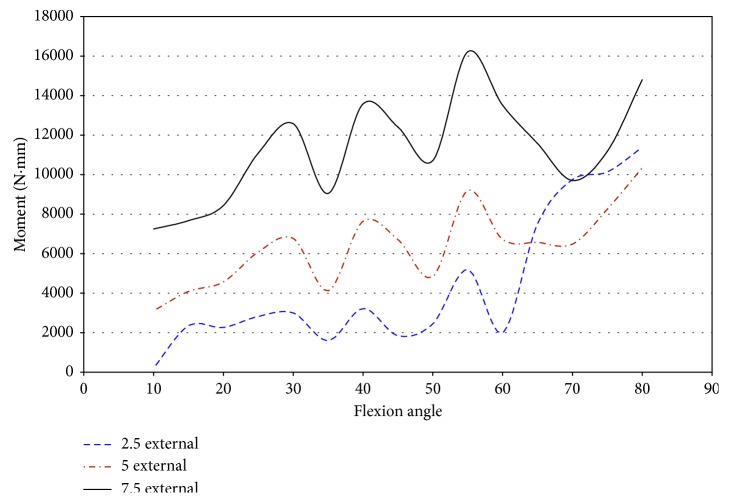
Magnitude of moments measured with JR3 UFS vs flexion angle; external rotation 43155.

The torque on specimen 43155 increases from 55° to 60° of flexion and then decreases from 60° to 70° of flexion during 5° and 7.5° external rotation (Figure 23). The torque increases from 60° to 70° of flexion for 2.5° of external rotation, contrary to the changes in torque for greater angles of external rotation. The net torque, during 7.5° external rotation, on the knee reaches a maximum at 55° flexion. This increase was preceded and followed by a decrease in net force at other flexion angles in which impingement was measured. The large change in moments when impingement was measured eliminates a trend of increased torque when the specimen experienced impingement. This lack of correlation between increased torque and increased force of impingement decreases the possibility of impingement contributing to injury of the ACL.

The maximum impingement forces range from 6.3 N to 11.67 N, which was substantially less than the amount of force the specimen incurred during loading. When considering that the net torque on the knee was at a maximum of approximately 16 N-m during impingement, the force of impingement seems even more inconsequential. The change in force and torque was inconsistent with the impingement forces. There appears to be no relationship between the force exerted on the knee and the impingement force.

### 5.6. JR3 Universal Force Sensor Measurements under Pure Valgus Rotation

During pure valgus, impingement was measured only in female specimens 50067 and 50108. In both of the female specimens, the analysis of force and torque measured by the JR3 UFS allows for a more in-depth understanding of the force acting on the knee, during flexion angles in which impingement occurred.

**Figure 24:**
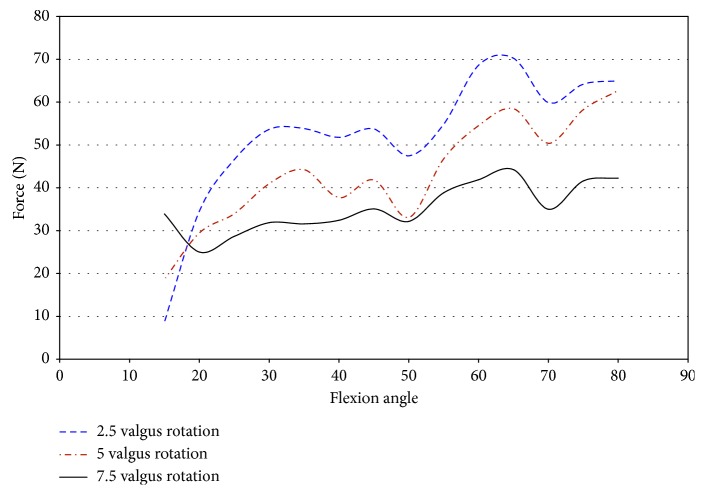
Magnitude of force measured by JR3 UFS vs flexion angle; valgus rotation 50067.

Impingement occurred at 15° of flexion in specimen 50067 during pure valgus rotation. This was the starting position of knee movement. This affects the ability to draw accurate conclusions on the effect impingement has on ACL injury in this specimen. The force was higher than the other specimens at the initial position. The entire range of net force and torque was plotted against the flexion angle to analyze for trends of increased loading on the knee due to impingement. The magnitude of force, shown in Figure 24, ranged from 8.7 N to 33.8 N at 15° flexion. At 7.5° valgus, the magnitude of force was approximately 34 N at 15° and 25 N at 20° flexion. The impingement force may affect the force exerted by the robot to complete the movement from 5° to 7.5° valgus. An indication that the impingement force was measured with the JR3 UFS was not found in any other specimen. The corresponding decrease was close to most of the impingement forces during this movement.

**Figure 25:**
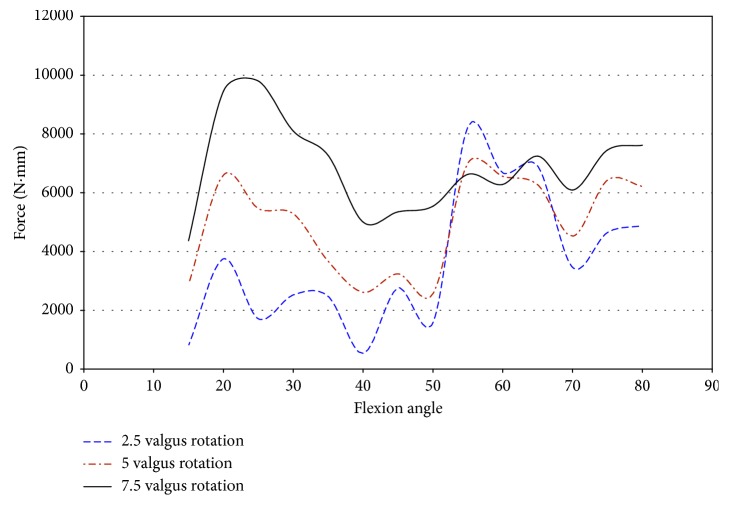
Magnitude of moments measured with JR3 UFS vs flexion angle; valgus rotation 50067.

During pure valgus rotation, in specimen 50067, the greatest torque occurs over the ranges of 20° to 25° of flexion and from 55° to 65° of flexion (Figure 25). Impingement occurred at 15° flexion. During pure valgus rotation, the moments measured were 4.4 N-m at 7.5° valgus rotation, 2.9 N-m at 5° valgus rotation, and less than 1 N-m for 2.5° valgus rotation all during 15° of flexion (Figure 25).

The absence of a large increase in torque, despite an increase in force during 7.5° of valgus, leads to the conclusion that, in this specimen, the impingement force has little or no effect on the healthy function of the knee. The significant increase in the amount of torque after 15° flexion could be significant if impingement also occurred at these flexion angles. In combined loading of 2.5°–7.5° valgus and 2.5°–7.5° external rotation, impingement was measured at these flexion angles making the scenario of unmeasured impingement occurring much less likely. Without a clear relationship between the force and torque increasing during impingement, the likelihood of ACL injury due to impingement was greatly reduced in this specimen.

In specimen 50108, impingement was measured from 15° to 35° of flexion.  Table 9 provides a summary of the impingement forces and measurements by the UFS. The strains never exceed 1.0%, but the torque measured by the UFS indicates that the knee was under considerable load. The force measured with the UFS is less than 10 N higher than the maximum impingement force at 15° flexion, approximately 13 N higher than the maximum impingement force at 35° flexion, and less than 2 N higher than the maximum impingement force at 30° flexion. It is important to consider the maximum torque during loading, which was substantially higher.  Table 9 shows the force of impingement was small when compared to the torque on the knee at the flexion angles in which impingement occurred. The strain was mostly compressive indicating resistance to valgus torque was from the MCL and not the ACL.

### 5.7. JR3 Universal Force Sensor Measurements under Valgus and External Rotation

In combination, loading impingement occurred in all female specimens and two of the three male specimens (though it should be noted that, in male knee 45492, the measurement consisted of very few points). The JR3 UFS again measured the force on the knees during these movements, and each knee has specific characteristics regarding the magnitude of force and torque when impingement was measured.

**Figure 26:**
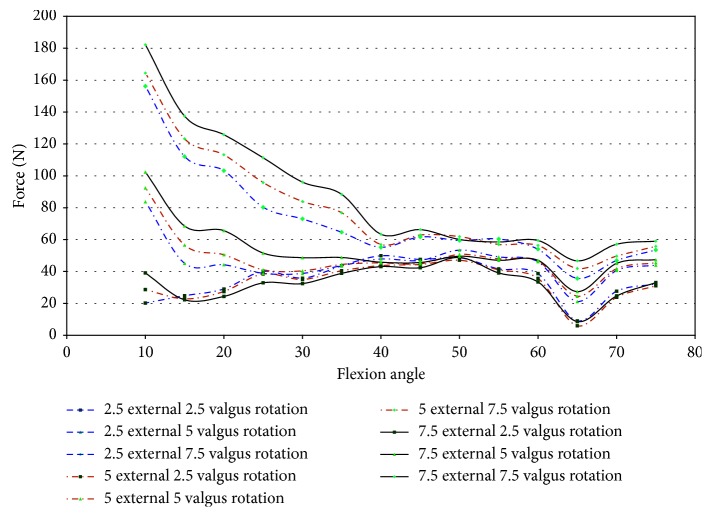
Magnitude of force measured with JR3 UFS vs flexion angle; combined loading valgus and external rotation 46921.

**Figure 27:**
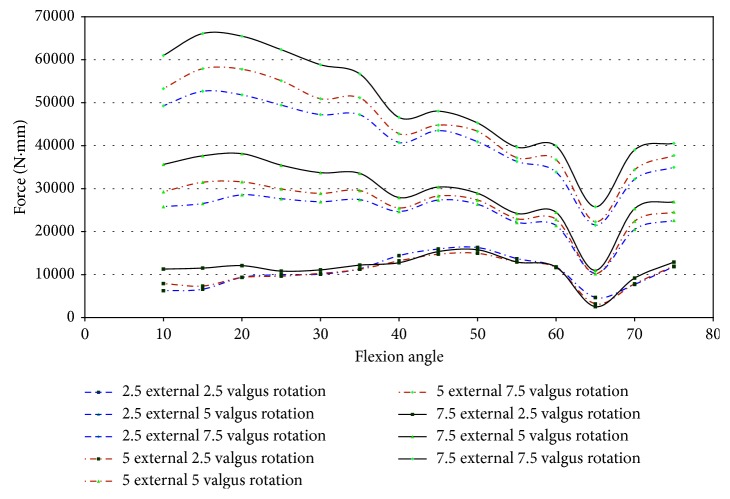
Magnitude of moments measured with JR3 UFS vs flexion angle; combined loading valgus and external rotation 46921.

Specimen 46921 experienced impingement between 10° and 45° of flexion, and at this range, the force decreased in all conditions of the knee. In all positions but 2.5° valgus combined with external rotation, the point of 10° flexion was the region with the greatest net force on the knee. It is apparent from Figure 26 that the force created from 2.5° to 7.5° of external rotation during 7.5° valgus rotation was a significant increase from the other movements. The significant increase at 7.5° valgus torque was important because this increase in the amount of net force indicates that the impingement force is small when compared to the measurements taken by the UFS. During flexion (10°–45°), when impingement occurred, the torque on the knee showed a similar relationship to the force on the knee, except near 15° and 20° flexion. At 15° and 20° of flexion, there was an increase in magnitude of torque (Figure 27). There was not a correlation in specimen 46921 between the net force and torque on the knee and the impingement force. The impingement force remained relatively constant at 10°, 20°, 25°, 35°, and 40° of flexion and decreased at 15° and 45° of flexion. The magnitude of force and torque measured by the UFS consistently decreased as the flexion angle increased from 10° to 45°. The force and torque increases from 40° to 45° of flexion. The elevated force and torque during the movement were likely a result of the PCL and MCL resisting external rotation and valgus torque.

In specimen 45492, impingement was measured at 45° of flexion.  Table 10 shows the impingement force was significantly less than the net force and the moments on the knee during flexion angles in which impingement occurred. The AM portion of the ACL experiences little strain, and thus the maximum torque on the knee was likely caused by the MCL-resisting valgus torque and PCL-resisting external torque. The force and torque on this specimen during impingement was less than the force and torque measured in specimen 46921, and the disparity was not large enough to indicate it was anything other than a difference in donors.

**Figure 28:**
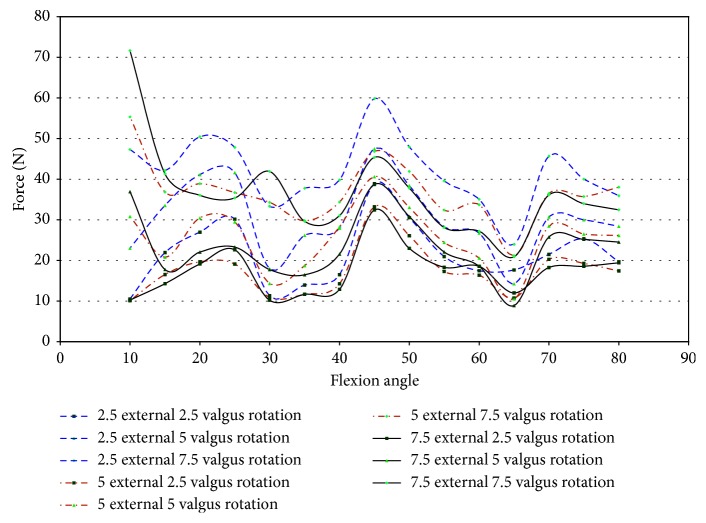
Magnitude of force measured with JR3 UFS vs flexion angle; combined loading valgus and external rotation 50108.

**Figure 29:**
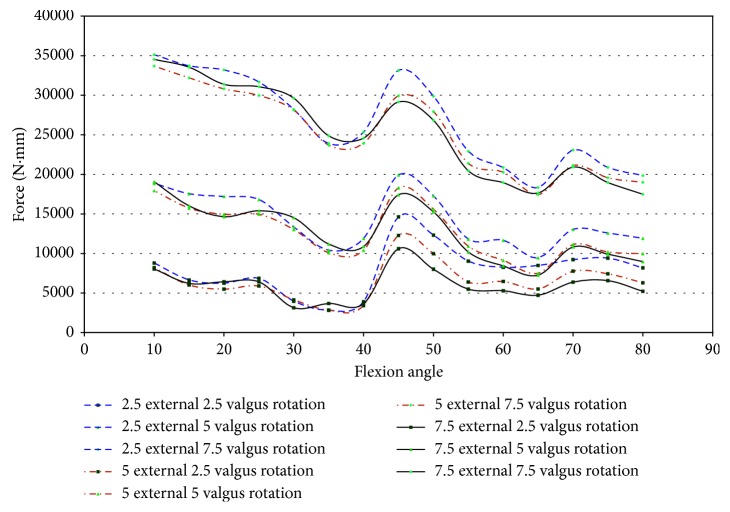
Magnitude of moments measured with JR3 UFS vs flexion angle; combined loading valgus and external rotation 50108.

Specimen 50108 had measured impingement at 15°–25° of flexion during combined loading of valgus and external rotation. The magnitude of force, shown in Figure 28, on the knee during these flexion angles varies greatly during each position. The force measured by the JR3 UFS during impingement was less in magnitude than the other specimens tested. The net torque on specimen 50108 during combined movement decreased during impingement (Figure 29). During combined movement of 7.5° valgus and external rotation, the torque on the knee increased from other movements tested, thus showing that the stress on the knee joint increases greatly past 5° valgus. The increased net force and torque were due to the increased strain in the ligaments of the knee.

When loading specimen 50067 from 2.5° to 7.5° valgus combined with 2.5° to 7.5° external rotation, impingement was measured during 15° and 20° of flexion. The contact of the DVRT with the notch roof created larger compressive strain than other specimens, but the increase in strain in this specimen was still small. When comparing the maximum impingement force to the maximum force and torque on the knee, the effect of impingement was small (Table 11).

**Figure 30:**
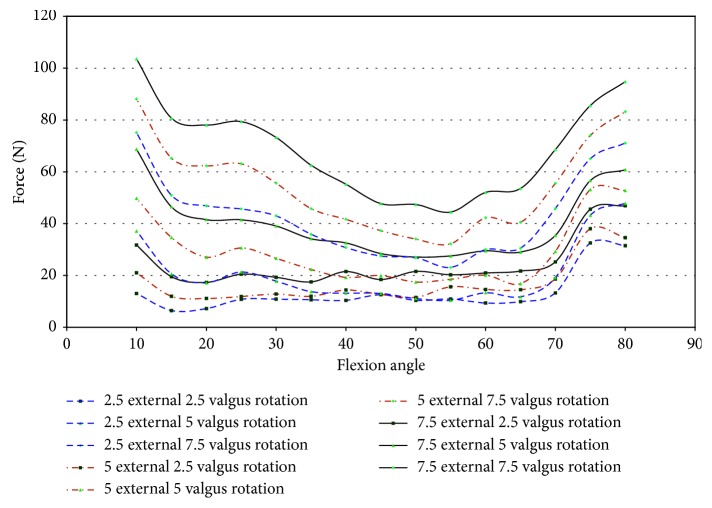
Magnitude of force measured with JR3 UFS vs flexion angle; combined loading valgus and external rotation 43155.

In specimen 43155, impingement was measured from 45° to 70° of flexion during 2.5°–7.5° valgus combined with 2.5°–7.5° external rotation.  Figure 30 shows the net force on the knee experiences a significant decrease during impingement. The lack of increased force when impingement was measured indicates that the contact between the ACL and the intercondylar notch wall has little or no effect on the function of the knee.

The torque on specimen 43155 (Figure 30) shows a similar decrease in force during the flexion angles in which impingement occurred (45°–70°). There was a large disparity between 7.5° valgus and 7.5° external rotation and other movements, but this was not uncommon as determined from the torque characteristics in other specimens during similar movements.

**Figure 31:**
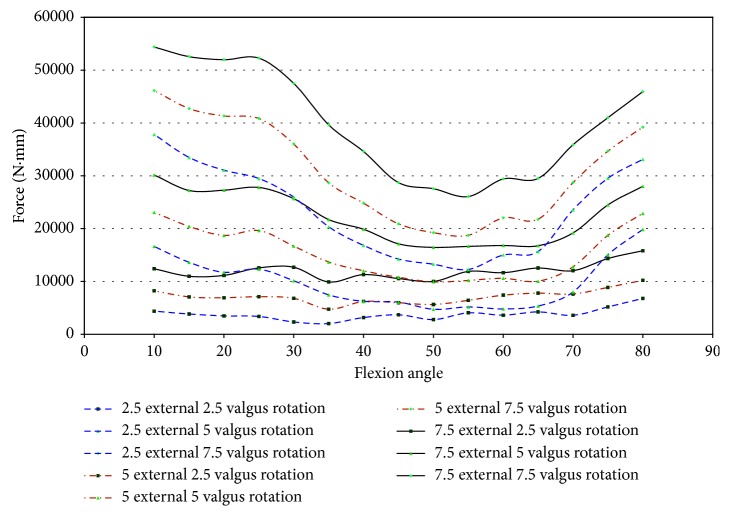
Magnitude of moments measured with JR3 UFS vs flexion angle; combined loading valgus and external rotation 43155.

The lack of increased force or increased moments when impingement was measured indicates impingement does not affect the healthy function of the knee in specimen 43155. The force and torque on the knee were due to the strain in the PCL and MCL during loading. The flexion angles impingement occurred do not correlate to areas of increased force on the knee. The force of impingement reached a maximum of 11.67 N at 65° flexion. This was considerably less than the maximum magnitude of force (53.57 N) and maximum magnitude of torque (29.5 N-m) at 65° flexion (Figure 31).

### 5.8. Analysis of Notch Geometry

The intercondylar notch geometry of each specimen is important for determining if a smaller intercondylar notch creates greater impingement forces. The measured characteristics of each notch are shown in Tables 2 and 3. The most important characteristics measured were the notch width at the exit, the notch width at the 2/3^rd^ notch height, and the bicondylar width. The notch width index (NWI-E) is based on the total notch width at the exit divided by the total bicondylar width. The notch width index at the 2/3^rd^ notch height (NWI-2/3^rd^) was calculated using the bicondylar width and the notch width at the 2/3^rd^ notch height (Table 2). None of the specimens in this study were below critical values of intercondylar notch stenosis, 0.2 for males, and 0.18 for females, as determined by Souryal and Freeman [3]. The minimum width of the ACL was also measured for reference to the size of the ACL in each of the specimen, specifically if ACL size could affect impingement. There is no relationship between the notch width exit and impingement with regards to all donors studied. Female specimens had smaller notch width exit measurements than male specimens. Specimen 50137 had the smallest notch width at exit, and impingement was not measured.

Notch width at the 2/3^rd^ notch height was less in female donors than in male donors. Donor 50137 had the smallest notch width at the exit and smallest notch width at the 2/3^rd^ notch height. Impingement was not measured in specimen 50137. Specimen 43155 had the smallest notch width at the exit and notch width at the 2/3^rd^ height. Smaller impingement forces and larger strains were found in 43155 than the other donors. The largest impingement forces for donors 46921 and 50108 were similar despite a variation in the notch width at the exit and notch width at the 2/3^rd^ notch height. It should be noted that female specimen 43155 had a build-up osteophytes along the notch roof making measurement of the notch width at the 2/3^rd^ height difficult and thus leading to a much smaller width.

The minimum width of the ACL for specimen 50137 was much smaller than the other male specimens. A significantly smaller ACL might reduce the occurrence of impingement, due to the ACL being a greater distance from the lateral notch wall. This would increase the probability of injury, since the ligament would have a lower mechanical threshold. It should be noted that the sample size of this study is not large enough in comparison with other research of this nature to make definitive conclusions regarding ACL injury, due to intercondylar notch geometry. These results should only be used as a reference relative to the experimental data obtained in this research.

The female specimens have smaller width at the notch width exit. The female specimens also had a smaller notch width index and notch width index at the 2/3^rd^ notch height, which was expected. The smallest knee, specimen 50067, had the smallest range of measured impingement among the female specimen, and a higher NWI than male specimen 50137, which was one in which no impingement was measured. Specimen 50108 has the largest notch width of female knees at the 2/3^rd^ notch height, at the notch exit, the second largest bicondylar width, and subsequent NWI larger than the one male knee (50137) that did not experience any impingement. Smaller measurements of the intercondylar notch, particularly the notch width exit, notch width at the 2/3^rd^ notch height, NWI (NWI-E), and NWI-2/3 have been shown to correlate to an increase in ACL injury in multiple studies [2, 3, 43, 44]. There was not a correlation between the measurements taken and impingement occurring in any of the specimen. The specimens with higher NWI-2/3 experienced more infrequent impingement. The measurements of the notch geometry may be a good indicator of increased probability for injury, but not a good indicator for the occurrence of impingement. It appears that the likelihood of impingement is caused by some other factors in notch geometry, likely the distance from the ACL to the lateral notch wall. A lack of correlation between the intercondylar notch measurements and the occurrence of impingement decreases the possibility that impingement is responsible for injury.

### 5.9. Discussion of Results

In male specimens tested, impingement is an insignificant factor in ACL injury. The corresponding increase in strain due to impingement was less then 1.28%. The male specimens did not show a drastic increase in strain during impingement. The forces of impingement in male specimens were similar in magnitude to female specimens but were more infrequent. In male knees, to achieve noticeable impingement, large levels of valgus and external rotation are required. This could be due to the compressive state of the ACL not being able to sustain contact. It may also be due to the distance of the ACL from the femoral condyle in male specimens. From the data, the large angles of valgus movement combined with external rotation were responsible for the increase in strain found in the anteromedial bundle of the ACL, and not the impingement force. The increase in valgus and external rotation could lead to greater impingement forces, but the strain due to these movements would also increase, thus increasing the probability of injury by compromising the structural integrity of the ACL.

Impingement is a product of valgus and external rotation, and in the male specimens tested, impingement would have little or no effect on ACL injury. The strain during the loading of the knee showed similar results to previous studies completed by Berns et al. [46] and Fleming et al. [47]. The strain measured during impingement was significantly less than the three-dimensional simulation completed by Fung and Zhang [6]. They calculated strains are close to 5% in the ACL, in their FE model. The corresponding impingement force under experimental conditions was 24 N. The calculated strain and impingement force was under larger levels of valgus and external rotation. Fung and Zhang calculated strain at approximately 1.0% under 7.5° valgus and 5° external rotation. Experimental results show the strain in the AM portion of the ACL in male specimens never exceeds 1.3%. None of the male specimens had an abnormally small notch width index, but the male specimen with the smallest notch width index was the only specimen measured without impingement. Notch width index at the 2/3^rd^ notch height does show some correlation to the occurrence of impingement, and the male specimens with larger notch width index at the 2/3^rd^ notch height had little or not any measured impingement. Specimen 50137 had a smaller minimum ACL width than male counterparts, which show the size of the ACL may affect impingement. A smaller ACL would have an increased distance to the lateral notch wall.

The force measurements taken by the JR3 UFS did not indicate that impingement increases the force or torque on the knee. The subsequent changes in force and torque with respect to the flexion angle are a function of the specific knee tested and are due to the increase in strain in the ligaments of the knee.  Table 12 is a summary of the maximum impingement force, flexion angle in which the maximum impingement force occurs, the maximum strain during impingement, and the maximum strain in a loading without impingement.

The female specimens were not as conclusive as the male specimens tested. There were many examples of disparity between the sexes with respect to the loading scenario. The magnitude of impingement force during pure external rotation was greater in female specimens (16.3 N and 19 N) than the male specimen 46921 (7 N). The magnitude of impingement force in female specimens was consistent with the male specimens during combination loading. The female specimens experienced impingement during pure valgus rotation, while there was not a male specimen with impingement during valgus torque. Female specimens show a larger increase in strain during loading of the knee and show a larger increase in strain during impingement.

The ACL in women has a lower modulus of elasticity than men and subsequently deforms more under the same force [41]. This provides an explanation for the increase strain in female specimens, but this does not explain a greater increase in strain during impingement. The change in strain during impingement is small, and with female specimens, there is less than 3% increase in strain during loading with impingement. The comparisons of strains are not between the same movements. The lack of impingement in a female specimen during combined valgus and external rotation leaves the study without a direct comparison of strain with identical movement. This is important, since the measurement of strain in 50137 during combination movement revealed a maximum strain of 2.2% and indicates that greater strains can be found in male specimens when impingement does not occur.

According to Berns et al. [46], valgus torque creates more strain than external rotation, and thus combined valgus and external rotation creates more torque than pure valgus. Using this information, these experimental results show impingement would have little or no effect on the strain in the ACL. The increase in strain would have resulted from the different movements applied to the knee. If impingement does contribute to increasing the strain in the AMB of the ACL, the increase is less than 3%. Fleming et al. [47] found that compressive load to the knee resulted in negligible increases in strain in the ACL. The studies by Berns et al. [46] and Fleming et al. [47] help support the conclusion that the strain increased due to impingement (if it occurs) would not increase under the force of bodyweight.

Specimen 43155 had a great disparity in strain in the anteromedial portion in comparison to the other specimens tested. This increase in strain can be explained using two specific reasons. The modulus of elasticity of the anterior cruciate ligament varies greatly from person to person [41]. The ACL in 43155 could have significantly lower modulus of elasticity than the other specimens tested. The tensile properties of the ACL have been known to vary with age. One study completed by Noyes and Grood [48] and separate research completed by Woo et al. [49] concluded that the tensile properties decrease with age, and both studies reported a decrease in the ultimate load of at least 50% for older specimens. Specimen 43155 was the oldest donor tested during this experiment and would have been grouped with the oldest specimens tested in the previously mentioned studies. Impingement forces, in specimen 43155, occurred at 45° to 70° of flexion. McNair et al. [50] and Boden et al. [51] reported the majority of ACL rupture occurred between 20° of flexion and full extension. In specimen 43155, the consequences in increased strain due to impingement are not significant due to the flexion angle that impingement occurred. The abovementioned research also supports the discounting of injury due to impingement in specimen 45492.

Female knee 50067 showed a correlation of increases in magnitude of force measured by the UFS during impingement. This does not specifically mean ACL impingement causes an increase in force or torque on the knee. This was the only example of a difference in force measured by the JR3 that corresponded to a change in impingement force. The decrease in force from 15° to 20° during pure valgus rotation corresponds to most of the impingement forces. The change in force compared to the change in the flexion angle for this specimen could be a natural function of the knee.

## 6. Conclusion

The experimental data show that impingement occurred in five of the six knees tested indicating it may be a regular occurrence, rather than a contributor to injury. The positions of the knee that create impingement largely increase the strain in the MCL and PCL, not the ACL. The addition of the bodyweight increases the force on the knee. Bodyweight also helps reduce the strain in the ACL by providing compression to the joint. Using in vitro testing methods, the addition of a body weight force would negate the effect muscle tissues have on normal knee function. It is also important to remember that impingement should still occur during these same movements. The measurement of strain during impingement provides a good reference of the increase in stress in the ACL during impingement. It still needs to be investigated if impingement is a factor in stenotic knees.

Given the low number of samples, a study with a larger number of samples would allow for more accurate conclusion regarding notch size and the differences between the genders for impingement of the ACL. A topography measurement of the distance from the ACL to the lateral notch wall through the full range of flexion could indicate individuals that have an increased possibility of impingement. Technological advances and continued testing that would allow for greater understanding of impingement as a noncontact mechanism of anterior cruciate ligament injury can be obtained, thus leading to better treatments of ACL injury and to better preventative measures used to mitigate ACL injuries.

## Figures and Tables

**Figure 32:**
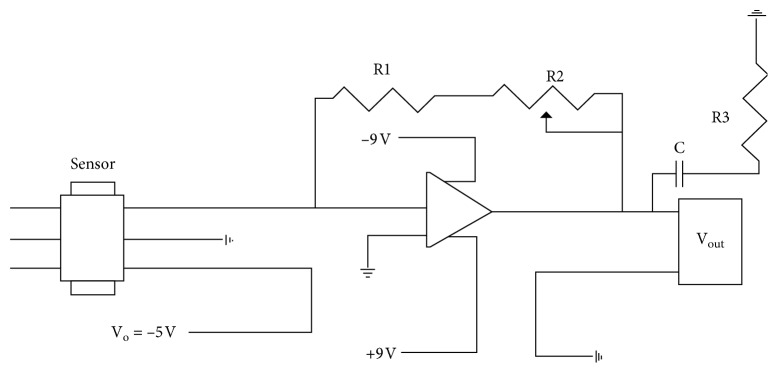
Conditioning circuit: R1 = 0.47 Ω; R2 = 20 KΩ; R3 = 10 Ω; C = 470 *μ*F.

**Figure 33:**
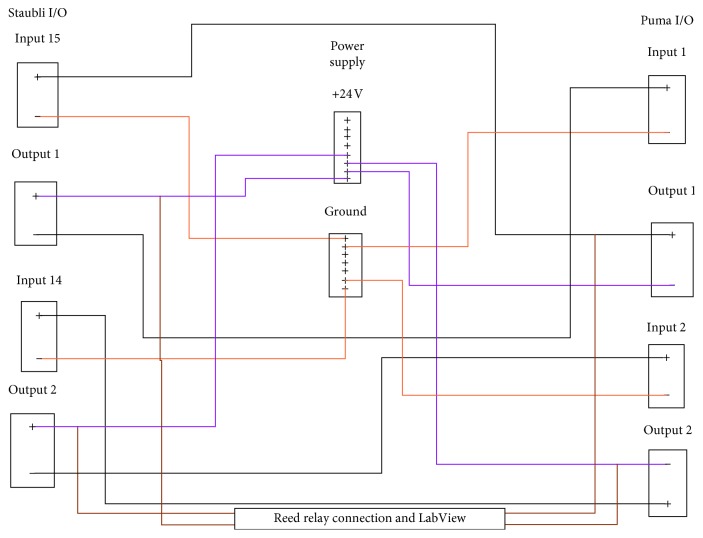
Robot input output control circuit.

**Table 1:** Age and anthropometry data of donors.

	Mean age (range), years	Mean body mass (range), kg	Mean height (range), cm	Mean body mass index (range), kg/m^2^	Mean lean body mass (range), kg
Male (*n* = 3)	58 (52–62)	81.80 (54.43–96.16)	180.34 (175.26–187.96)	24.98 (17.72–30.00)	62.56 (47.53–72.27)
Female (*n* = 3)	49.67 (38–66)	82.55 (54.43–113.40)	158.32 (154.94–160.02)	33.22 (21.26–47.24)	43.92 (41.12–48.58)

**Table 2:** Notch geometry of specimens.

Specimen	Knee	Gender	Notch width at the exit (mm)	Notch width at the 2/3^rd^ notch height (mm)	Notch height (mm)	Bicondylar width (mm)	Minimum ACL width (mm)
43155	Left	Female	16.129	6.48	20.07	75.44	12.94
45492	Left	Male	21.02	19.926	30.66	87.15	17.08
46921	Right	Male	21.14	14.74	30.2	82.6	15.99
50067	Right	Female	14.33	9.68	19.62	57.84	10.56
50108	Right	Female	16.83	13.21	23.65	71.7	10.16
50137	Left	Male	18.69	15.55	33.53	80.62	12.13

**Table 3:** Notch width indices of specimens.

Specimen	Knee	Gender	NWI-E	NWI-2/3^rd^
43155	Left	Female	0.213799	0.085896
45492	Left	Male	0.241193	0.22864
46921	Right	Male	0.255932	0.17845
50067	Right	Female	0.247752	0.167358
50108	Right	Female	0.234728	0.18424
50137	Left	Male	0.231828	0.19288

**Table 4:** Knee movements.

Flexion angle	Varus and valgus rotation angles				
	0°	2.5°	5°	7.5°	
0°	2.5°, 5°, 7.5°	2.5°, 5°, 7.5°	2.5°, 5°, 7.5°	2.5°, 5°, 7.5°	Internal and external rotation angles
5°	2.5°, 5°, 7.5°	2.5°, 5°, 7.5°	2.5°, 5°, 7.5°	2.5°, 5°, 7.5°	
10°	2.5°, 5°, 7.5°	2.5°, 5°, 7.5°	2.5°, 5°, 7.5°	2.5°, 5°, 7.5°	
15°	2.5°, 5°, 7.5°	2.5°, 5°, 7.5°	2.5°, 5°, 7.5°	2.5°, 5°, 7.5°	
20°	2.5°, 5°, 7.5°	2.5°, 5°, 7.5°	2.5°, 5°, 7.5°	2.5°, 5°, 7.5°	
25°	2.5°, 5°, 7.5°	2.5°, 5°, 7.5°	2.5°, 5°, 7.5°	2.5°, 5°, 7.5°	
30°	2.5°, 5°, 7.5°	2.5°, 5°, 7.5°	2.5°, 5°, 7.5°	2.5°, 5°, 7.5°	
35°	2.5°, 5°, 7.5°	2.5°, 5°, 7.5°	2.5°, 5°, 7.5°	2.5°, 5°, 7.5°	
40°	2.5°, 5°, 7.5°	2.5°, 5°, 7.5°	2.5°, 5°, 7.5°	2.5°, 5°, 7.5°	
45°	2.5°, 5°, 7.5°	2.5°, 5°, 7.5°	2.5°, 5°, 7.5°	2.5°, 5°, 7.5°	
50°	2.5°, 5°, 7.5°	2.5°, 5°, 7.5°	2.5°, 5°, 7.5°	2.5°, 5°, 7.5°	
55°	2.5°, 5°, 7.5°	2.5°, 5°, 7.5°	2.5°, 5°, 7.5°	2.5°, 5°, 7.5°	
60°	2.5°, 5°, 7.5°	2.5°, 5°, 7.5°	2.5°, 5°, 7.5°	2.5°, 5°, 7.5°	
65°	2.5°, 5°, 7.5°	2.5°, 5°, 7.5°	2.5°, 5°, 7.5°	2.5°, 5°, 7.5°	
70°	2.5°, 5°, 7.5°	2.5°, 5°, 7.5°	2.5°, 5°, 7.5°	2.5°, 5°, 7.5°	
75°	2.5°, 5°, 7.5°	2.5°, 5°, 7.5°	2.5°, 5°, 7.5°	2.5°, 5°, 7.5°	
80°	2.5°, 5°, 7.5°	2.5°, 5°, 7.5°	2.5°, 5°, 7.5°	2.5°, 5°, 7.5°	
85°	2.5°, 5°, 7.5°	2.5°, 5°, 7.5°	2.5°, 5°, 7.5°	2.5°, 5°, 7.5°	
90°	2.5°, 5°, 7.5°	2.5°, 5°, 7.5°	2.5°, 5°, 7.5°	2.5°, 5°, 7.5°	

**Table 5:** FlexiForce® sensor properties.

Sensor properties	Model A202-25
Operating range	−9°C to 60°C
Linearity (error)	<+/− 5%
Repeatability	<+/− 2.5% of full scale
Hysteresis	<+/− 4.5% of full scale
Drift	<3% of logarithmic time scale
Temperature sensitivity	Variance of 0.36% per °C

**Table 6:** Summary of impingement.

Specimen	Knee	Gender	Flexion pure external rotation	Flexion pure valgus rotation	Flexion valgus and external rotation
45492	Left	Male	N/A	N/A	45°
46921	Right	Male	20°, 40°	N/A	10°, 15°, 20°, 25°, 35°, 40°, 45°
50137	Left	Male	N/A	N/A	N/A
50108	Right	Female	50°	15°, 20°, 25°, 30°, 35°	15°, 20°, 25°
43155	Left	Female	50°, 55°, 60°, 65°	N/A	45°, 50°, 55°, 60°, 65°, 70°
50067	Right	Female	N/A	15°	15°, 20°

**Table 7:** Impingement summary and JR3 UFS measurements for the male specimen 46921; external rotation.

Flexion angle impingement	Range of strain during impingement (% strain)	Range of impingement force (N)	Range of magnitude of force during impingement measured by JR3 UFS (N)	Range of magnitude of moments during impingement measured by JR3 UFS (N-m)
20°	0.07–0.784	1.91–7.05	25.7–26.5	4.4–7.0
40°	0.53–0.62	2.79–4.19	40.2–45.0	7.0–11.5

**Table 8:** Impingement summary and JR3 UFS measurements for the male specimen 50108; external rotation.

Flexion angle impingement	Range of strain during impingement (% strain)	Range of impingement force (N)	Range of magnitude of force during impingement measured by JR3 UFS (N)	Range of magnitude of moments during impingement measured by JR3 UFS (N-m)
50°	−0.9–−0.63	1.28–19.02	28.2–41.9	10.5–17.5

**Table 9:** Impingement summary and JR3 UFS measurements for male specimen 50108; valgus rotation.

Flexion angle impingement	Range of strain during impingement (% strain)	Range of impingement force (N)	Range of magnitude of force during impingement measured by JR3 UFS (N)	Range of magnitude of moments during impingement measured by JR3 UFS (N-m)
15°	−1.57 to 0.91	0.62 to 21.89	20.56 to 30.65	2.38 to 18.15
20°	−1.47 to −0.13	1.73 to 15.66	24.87 to 39.30	3.07 to 16.90
25°	−1.29 to 0.05	1.73 to 14.49	22.51 to 37.48	2.00 to 17.05
30°	−1.62 to −0.32	0.33 to 16.91	11.8 to 18.65	1.97 to 14.21
35°	−1.27 to −0.48	0.41 to 10.1	14.73 to 23.32	1.32 to 11.10

**Table 10:** Impingement summary and JR3 force measurements for male specimen 45492; valgus and external rotation.

Flexion angle impingement	Range of strain during impingement (% strain)	Range of impingement force (N)	Range of magnitude of force during impingement measured by JR3 UFS (N)	Range of magnitude of moments during impingement measured by JR3 UFS (N-m)
45°	0.26–0.43	0.5–16.98	36.41–75.62	10.06–32.40

**Table 11:** Impingement summary and JR3 force measurements for female 50067; valgus and external rotation.

Flexion angle impingement	Range of strain during impingement (% strain)	Range of impingement force (N)	Range of magnitude of force during impingement measured by JR3 UFS (N)	Range of magnitude of moments during impingement measured by JR3 UFS (N-m)
15°	−6.70 to 4.10	0.294 to 14.37	5.56 to 51.67	2.26 to 10.30
20°	−3.99 to −3.92	2.64 to 9.39	20.16 to 31.01	5.12 to 14.24

**Table 12:** Maximum impingement and strain.

Specimen number	Gender	Magnitude of forces measured by JR3 UFS (N)	Loading and flexion angle	Maximum impingement force (N)	Maximum strain (%)	Maximum strain without impingement (%)
43155	Female	|F| = 29.00; |M| = 11.60	Pure external 65°	16.86	7.05	4.90
45492	Male	|F| = 75.62; |M| = 32.40	Valgus and external 45°	16.98	0.43	0.25
46921	Male	|F| = 88.46; |M| = 56.70	Valgus and external 35°	19.65	1.28	0.46
50067	Female	|F| = 33.88; |M| = 4.37	Pure valgus 15°	24.39	4.07	1.10
50108	Female	|F| = 30.66; |M| = 18.15	Pure valgus 15°	21.89	1.68	N/A
50137	Male	N/A	N/A	N/A	N/A	2.20

## Data Availability

The data are included in the charts that are provided. The data were collected directly via computer-based data acquisition equipment.

## Appendix

### FlexiForce® Sensor Conditioning Circuit

A schematic of the FlexiForce® conditioning circuit is shown in Figure 32. This circuit was connected to LabView to trigger the recording of data from the FlexiForce® sensor and DVRT. A reed relay was installed on a board, so the 24 V signals from the robot I/O circuits was reduced to 5 V provided by the same power source. The use of the 5 V signal, instead of the 24 V signal from the robots, to the LabView DAQ board prevents damage, since the board is only rated for a maximum of +/− 5 V. The interface circuit was color coded as follows: black wire connected robot to robot, green wire connected to the ground, purple wire connected to +24 V terminal of the power supply, and for the first I/O circuit, blue wire connected the first set of output signals (one from the Puma and one from the Staubli) to the reed relay that is connected to the LabView DAQ board (Figure 33).

For the second I/O circuit, white wire connected the second set of output signals to the reed relay for connection to LabView. A resistor of 1 KΩ was installed in series with each of the grounds of the relays for the I/O with the data acquisition board of LabView. This allows for the switch to close (creating a 5 V digital signal to LabView) but does not pull enough current to harm the robot controllers or the LabView DAQ board. The amount of current was approximately 10 mA, the Staubli I/O is rated for 20 mA, and the Puma I/O is rated for 0.5 A. For the DAQ board to recognize the digital trigger, it must be TTL compliant. This implies the rise time on the signal must be less than 50 nanoseconds, and the relays used in this setup were Radio Shack model 275–232 reed relays, which are TTL compliant.

## Supplementary Materials

Supplementary Materials Provided in the supplemental materials is an MPEG 4 video capturing the combined robot movements during the testing of one of the knee specimens. Seen in the video is the roll-glide motion described in the body of the paper.
